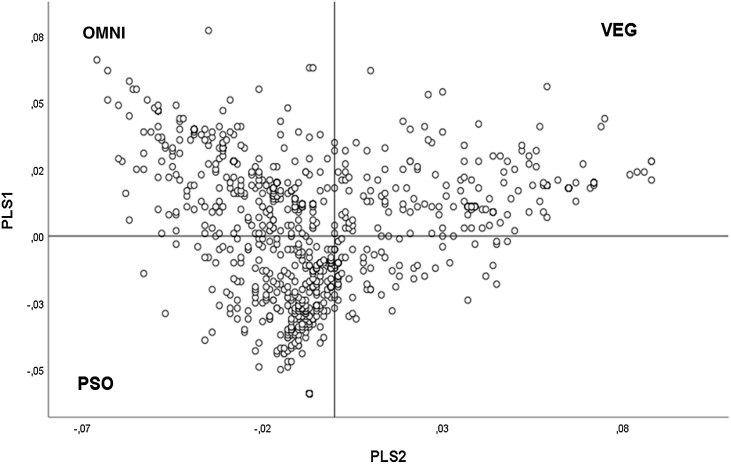# Intestinal microbiome characterization of adult Brazilian men with psoriasis compared to omnivore and vegetarian controls^☆^

**DOI:** 10.1016/j.abd.2022.08.008

**Published:** 2023-05-06

**Authors:** Tatiana Cristina Figueira Polo, Mariana Righetto de Ré Lai, Luciane Donida Bartoli Miot, Giovana Fernanda Cosi Bento, Márcia Guimarães da Silva, Silvio Alencar Marques, Hélio Amante Miot

**Affiliations:** a Department of Dermatology, Botucatu Faculty of Medicine, Universidade Estadual Paulista, Botucatu, SP, Brazil; b Department of Pathology, Botucatu Faculty of Medicine, Universidade Estadual Paulista, Botucatu, SP, Brazil

**Keywords:** Diet, Western diet, Gastrointestinal microbiome, Microbiota, Obesity, Psoriasis, Vegetarian diet

## Abstract

**Background:**

Psoriasis is a chronic inflammatory disease associated with systemic inflammation and comorbidities. Changes in the composition of the intestinal microbiome are involved in the pathogenesis of inflammatory diseases and metabolic syndrome. Characterizing the intestinal microbiome of patients with psoriasis may be relevant for the understanding of its clinical course and comorbidity prevention.

**Objective:**

To characterize the intestinal microbiome of men with psoriasis compared to omnivore and vegetarian controls (without psoriasis).

**Method:**

Cross-sectional study of 42 adult males: 21 omnivores with psoriasis; and controls: 14 omnivores and 7 vegetarian individuals. The characterization of the intestinal microbiome was performed by metagenomic analysis. Serum levels of lipopolysaccharide-binding protein (LPB) and C-reactive protein (CRP) were evaluated.

**Results:**

The groups differed from each other regarding nutritional aspects and microbiome; individuals with psoriasis had a higher consumption of protein and lower consumption of fibers. Levels of LPB, CRP, and the *Firmicutes/Bacteroidetes* ratio were higher in the group with psoriasis than in the vegetarian group (p < 0.05). The genera *Prevotella*, *Mogibacterium*, *Dorea*, *Bifidobacterium* and *Coprococcus*, differed in the group with psoriasis compared to vegetarians; the genera *Mogibacterium*, *Collinsella* and *Desulfovibrio* differed from omnivores. A microbiome pattern linked to psoriasis (plsPSO) was identified, which was associated with higher LPB levels (rho = 0.39; p = 0.02), and lower dietary fiber intake (rho = −0.71; p < 0.01).

**Study limitations:**

Only adult men were evaluated.

**Conclusion:**

A difference was identified in the intestinal microbiome of adult men with psoriasis when compared to healthy omnivores and vegetarian controls. The identified microbiome pattern was correlated with dietary fiber intake and serum levels of LPB.

## Introduction

Psoriasis is a chronic systemic inflammatory disease, affecting 1% to 2% of the population.^1^ Its pathogenesis is multifactorial, and not yet fully understood, but it involves, in addition to a genetic basis and environmental stimuli, the activation of the immune system (innate and adaptive), which triggers local inflammatory responses in the skin and joints, as well as promoting low-grade systemic inflammation.^2^

Comorbidities such as obesity, arterial hypertension, diabetes mellitus, metabolic syndrome, cardiovascular disease, dyslipidemia, and affective disorders (depression, anxiety) are frequent in patients with psoriasis, corroborating the underlying systemic inflammatory process.^3^

The intestinal microbiome (IM) consists of an inconstant flora, established in the first years of life, but which varies according to environmental stimuli, such as diet, lifestyle (smoking, alcohol consumption, physical exercise, stress, sleep), hygiene, therapy with antimicrobials, geographic location, sex, age, and certain diseases. About 57% of the IM is influenced by diet and only 12% by genetic factors.^4^ There are between one and two thousand species of bacteria in the fecal microbiome, distributed in more than fifty phyla. About 90% of the IM consists of the *Firmicutes* and *Bacteroidetes* phyla, where a higher *Firmicutes/Bacteroidetes* (F/B) ratio is associated with a greater systemic inflammatory stimulus.^5^

Intestinal dysbiosis, characterized by IM composition imbalance, can lead to increased intestinal permeability and translocation of intraluminal inflammatory mediators. Therefore, the characterization of IM can contribute to the understanding of the pathogenesis of systemic inflammatory diseases, such as psoriasis, Crohn's disease, rheumatoid arthritis, and obesity, since there are indications that IM composition is involved in their course.^6^

Clinical studies have been conducted to explore the association between IM and psoriasis clinical activity with conflicting results.^7^ However, MI modulation through dietary treatment, supplementation with probiotics, and fecal transplantation is considered to have the potential to interfere with the course of comorbidities, in addition to psoriasis activity, as well as maximizing therapeutic response.^8^

Lipopolysaccharide (LPS) is an endotoxin that constitutes the cell wall of gram-negative bacteria and promotes the immune system activation by direct stimulation of macrophages, monocytes, dendritic cells and B lymphocytes, triggering fever, vasodilation (nitric oxide), and eicosanoid secretion.^9^ Lipopolysaccharide-binding protein (LPB) binds to LPS and transfers LPS monomers to inflammatory cells via CD14. Plasma levels of LPB are influenced by circulating levels of LPS, which have a very short half-life. Exposure to LPS induces a lasting increase in LPB production in the liver within 15 to 30 minutes, with a peak serum level occurring after 24 to 48 hours. The LPS-LBP-CD14-MD2 complex induces a pro-inflammatory response through toll-like-4 (TLR4) receptor-mediated NF-κB activation, and thus, circulating levels of LPB have been associated with systemic inflammation. Additionally, plasma levels of highly-sensitive quantitative C-reactive protein (hs-CRPqt), a serum biomarker of systemic inflammation synthesized by the liver, are associated with increased LPS.^10^

Controlled studies characterizing IM in patients with psoriasis are rare in South America. Therefore, the aim of this investigation was to characterize IM of Brazilian adult men with psoriasis, in comparison with omnivore and vegetarian controls.

## Method

This was a cross-sectional study involving 42 adult males (aged 21 to 65 years): 21 with psoriasis (PSO); and 21 controls without psoriasis: 14 omnivores (OMNI), and seven vegetarians (VEG).

All participants included in the study were non-smokers, non-alcoholics, had not changed their diet in the last three months; did not use antibiotics, laxatives, or anti-inflammatory drugs, or reported intestinal changes in the previous week, and did not have inflammatory bowel disease, infections, autoimmune diseases or used probiotics. The sample of adult men from the interior of the state of São Paulo was chosen to aim reducing MI variability, which is also influenced by sex, age, and geographic location of the population.

The study was approved by the Human Research Ethics Committee of the Botucatu Faculty of Medicine – FMB/UNESP (Counsel number 3,743,366); and all participants signed the Free and Informed Consent Form.

The study was carried out at the Dermatology Outpatient Clinic of Botucatu Faculty of Medicine, from March 2020 to February 2021. The population comprised individuals who lived in the area of Botucatu, the state of São Paulo, comprising patients diagnosed with psoriasis, their companions, and vegetarians who were invited to participate.

Clinical, demographic, socioeconomic, and dietary data were obtained through in-person interviews; anthropometric and dietary assessments were performed by an experienced nutritionist (T.C.F.P.).

The dietary assessment was obtained using the 24-h recall (R24 h) method, based on a detailed account of food consumption in the last 24 hours, including data on types of foods and beverages, in addition to sizes, portions, volume, or household measurements. Subsequently, data were transformed into grams, energy values, macronutrients, and micronutrients using the Nutritional Data System for Research (NDSR) software. To estimate disease severity, the PASI (Psoriasis Area and Severity Index) score was used, which was evaluated by a certified dermatologist (L.D.B.M. or M.R.R.L).

To assess IM, stool samples were collected to obtain the genetic material. The individuals were instructed to perform the stool collection in a specific tube in their homes, consisting of the first evacuation of the day. After collection, the stool samples were delivered to the researcher in charge (T.C.F.P.) and transported while refrigerated (dry ice: −28 °C) to the Biotechnology Research and Innovation Laboratory (BPI, *Biotecnologia Pesquisa e Inovação*, Botucatu-SP), and stored in a freezer at -80 ° C until the time of DNA extraction.

To extract the genetic material, 1 g aliquots and the Quick-DNA Fungal/Bacterial Miniprep kit (Zymo Research) were used according to the protocol described by the manufacturer. Subsequently, the material underwent a quality test through fluorescence quantification using the Qubit® 3.0 Fluorometer (Thermo Fisher Scientific) equipment.

Sample triplicates were used for amplification reactions of the 16S region, with a final volume of 20 μL, containing 10 μL of GoTaq® Colorless Master Mix 2× (Promega, USL), 0.3 μM of forward oligonucleotide and 0.3 μM of the reverse oligonucleotide, 3 μL of genomic DNA, and sufficient sterile ultrapure water to make up 20 μL. Amplification reactions were carried out in a Veriti™ Thermal Cycler (Applied Biosystems).

After the DNA amplification reaction of each sample, proof of amplification was performed through electrophoresis in 2% agarose gel stained with Gel Red (Uniscience), approximately 300 bp (amplicon size). PCRs were submitted to purification steps using Agencourt AMPure XP magnetic bead (Beckman Coulter), to remove very small fragments from the total population of molecules and remnants of primers from the reaction. After this step, the quantification was performed using Real-Time PCR methodology using QuantStudio 3 Real-Time thermal cycler (Applied Biosystems) and the KAPA-KK4824 kit (Library Quantification Kit ‒ Illumina/Universal), all according to the manufacturer protocol.

An equimolar pool of DNA was generated by normalizing all samples to 3 nM to perform sequencing, which was carried out using the Illumina MiSeq next-generation sequencing system (Illumina Sequencing) and the MiSeq Reagent Kit V2 Nano 300 cycles – reading of 2 × 150bp.

The Greengenes 13_8 99% OTUs database from 515 F/806R region of sequences was used to identify taxon sequences and only sequences with more than 97% analogy (reliability) were considered.

The relative abundance of taxonomic units (TU) in the samples was explored based on their components extracted by the PLS (Partial Least Squares) method, with data scaled by standard deviations.

The main study variables are the diversity of the intestinal flora identified in each evaluated IM, according to each taxonomic unit (TU) category: relative abundance of the groups, by the Hill diversity indices (richness, Shannon and Simpson), the ratio between the *Firmicutes/Bacteroidetes* phyla, the abundance of bacterial genera (alpha diversity), and the components of the PLS patterns (beta diversity), identified for the groups. Richness is defined as the total number of TU identified in the sample.

Samples of 10 mL of blood were also collected from patients at the dermatology outpatient clinic, by the physician responsible for treating the patients (M.R.R.L.) for the assessment of serum LPB and hs-CRPqt. After collection, the samples were immediately taken under refrigeration to the Experimental Research Unit (UNIPEX) of FMB-Unesp, for centrifugation and serum extraction. For LPB analysis, the ELISA (Enzyme-Linked Immunosorbent Assay) sandwich method was used, according to the kit manufacturer recommendations (Human LBP DuoSet ELISA ‒ DY870-05, R&D Systems, Inc., Minneapolis, MN, USA), with the samples being diluted at 1:500. Final concentrations of LPB were determined according to the standard curve. The CRPqt analyses were performed using the immunoturbidimetry method. Values of CRPqt ≤ 3 mg/dL and LPB ≤ 10 mg/mL were considered normal.

Qualitative variables were represented by percentages. Quantitative variables were represented as means and standard deviations if normality was verified by the Shapiro-Wilks test, or medians and quartiles (P25–P75) if indicated. The abundance of genera were compared between groups using the Kruskal-Wallis test. Ecological diversity indices were compared between groups using generalized linear models, adjusted for age. The models were adjusted to the most appropriate probability distributions for each sample (e.g., gamma), and correction for multiple comparisons was performed using the Šidák procedure.

The loads of PLS components were correlated with nutritional data, LPB levels, and hs-CRPqt by Spearman correlation test, and represented as a heat map. The data were tabulated in MS Excel 2010 and analyzed in IBM SPSS 25v and Statistica 10v software. Statistical significance was defined as p ≤ 0.05, two-tailed.

As this is an exploratory study, convenience sampling was used to determine the global behavior of the groups and support the detailed analysis of samples *a posteriori*. Moreover, the PLS method does not require a minimum sampling size to be performed. For the bivariate correlation analyses, a sample of 41 participants is considered sufficient to detect a moderate correlation (rho < 0.70), considering an α = 0.01 and power of 90%.

## Results

The main clinical, demographic, dietary data, serum LPB, and hs-CRPqt levels are shown in Table 1. Age and BMI of the PSO and OMNI groups were higher than those of the VEG group. From the dietary point of view, the PSO and OMNI groups reported higher consumption of carbohydrates and proteins than the VEG group. As for dietary fiber, the PSO group reported lower fiber intake than the OMNI and VEG groups. Plasma levels of LPB, as well as levels of hs-CRPqt, were higher in the PSO group in relation to the VEG group; however, hs-CRPqt levels were higher in the PSO group compared to the OMNI group.

**Table 1:** Main demographic and dietary data, serum LPB and hs-CRPqt levels of participants (n = 42)

	Omnivore controls (n = 14)	Vegetarian controls (n = 7)	Psoriasis	p-Value
Age (years)*	41.6 (11.3)^a^	25.4 (2.5)	51.1 (11.1)^a^	<0.001
BMI (kg/m^2^)*	31.1 (9.2)^a^	24.4 (2.5)	29.5 (6.2)^a^	<0.001
Energy (cal)*	3.859.4 (1297.5)^a^	2.314.3 (129.9)	3.058.0 (512.0)^a^, ^b^	<0.001
Carbohydrates (g)*	539.5 (185.7)^a^	362.2 (22.8)	466.9 (86.8)^a^	<0.001
Proteins (g)*	198.9 (76.0)^a^	58.4 (21.6)	133.4 (39.3)^a^, ^b^	<0.001
Lipids (g)*	101.2 (45.5)^a^	72.0 (9.7)	73.9 (23.2)	0.022
Fibers (g)*	21.1 (9.3)^a^	42.4 (7.1)	13.8 (7.4)^a^, ^b^	<0.001
LPB (mg/mL)*	6.1 (2.9)	4.8 (1.5)	7.6 (2.9)^a^	0.007
hs-CRPqt (mg/dL)*	2.6 (3.2)	1.0 (0.6)	5.2 (9.2)^a^, ^b^	0.003

* Mean(standard deviation); BMI, Body Mass Index; LPB, Lipopolysaccharide binding protein; hs-CRPqt, highly-sensitive C-reactive protein, quantitative test.

a p ≤ 0.05 – Compared to vegetarians.

b p ≤ 0.05 – Compared to omnivores.

No VEG controls or OMNI individuals with normal weight showed high LPB values; however, one obese OMNI individual, one PSO patient with normal weight, and two obese PSO patients showed LPB > 10 mg/mL. Similarly, no VEG or OMNI controls with normal weight showed high values of hs-CRPqt; however, two obese OMNI individuals, four PSO patients with normal weight, and three obese PSO patients showed CRP levels > 3 mg/mL.

Among patients with psoriasis, eight (32%) presented joint disease, six (24%) were treated exclusively with topical drugs, six (24%) used methotrexate, eight (32%) used acitretin, and five (20%) used secukinumab. The PASI score ranged from 1 to 18, with a median (P_25_–P_75_) of 4 (1–7).

As for the comorbidities, among patients with psoriasis, two (17%) had diabetes mellitus, three (25%) had arterial hypertension, and five (42%) had dyslipidemia. Among the omnivore controls, two (13%) had diabetes, four (28%) had arterial hypertension and six (43%) had dyslipidemia. None of the vegetarian controls had comorbidities.

The analysis of IM of the participants resulted in the identification of 1,496 different taxonomic units (TU), grouped into 94 genera (Supplementary). IM ecological diversity indices differed between the groups, as shown in Table 2. When adjusted for age and BMI, the phylum *Firmicutes* was less abundant in VEG, and the F/B ratio was higher in the PSO than in the VEG group.

**Table 2:** Ecological diversity indices of the intestinal microbiome amongst the groups (n = 42)

	Omnivore controls (n = 14)	Vegetarian controls (n = 7)	Psoriasis (n = 21)	p-Value^a^
Relative abundance (10^3^)^b^	62.0 (12.6)	63.7 (13.7)	63.6 (11.0)	0.989
Hill's Diversity^b^				
q = 0 (richness)	141.2 (53.6)	136.6 (21.1)	138.5 (49.8)	0.353
q = 1 (Shannon)	38.0 (18.0)	33.6 (15.2)	39.4 (18.1)	0.507
q = 3 (Simpson)	13.4 (6.7)	11.8 (7.3)	14.5 (7.5)	0.813
Abundance according to phylum^c^				
*Firmicutes* (10^3^)	40.1 (37.9–46.6)^d^	35.1 (29.6–37.0)	39.1 (33.1–48.8)^d^	**0.049**
*Bacteroidetes* (10^3^)	17.7 (6.5–23.1)	32.0 (10.9–40.4)	13.1 (6.8–23.5)	0.335
F/B ratio	2.5 (1.8–6.0)	1.0 (0.9–3.5)	3.1 (2.2–5.9)^d^	**0.045**

F/B, *Firmicutes/Bacteroidetes*.

a p-value adjusted for age and Body Mass Index.^b^ Mean (standard deviation); ^c^ Median (P_25_–P_75_).

d p ≤ 0.05 – Compared to vegetarians.

b p ≤ 0.05 – Compared to omnivores.

When comparing obese patients with psoriasis patients and obese controls, the F/B ratio showed no difference (p = 0.078). However, when individuals with psoriasis and normal weight were compared to controls with normal weight, patients with psoriasis had higher mean values (SD) of the F/B ratio (12.1 [2.9] vs. 2.0 [1.0]; p < 0.01).

**Figure 1:**
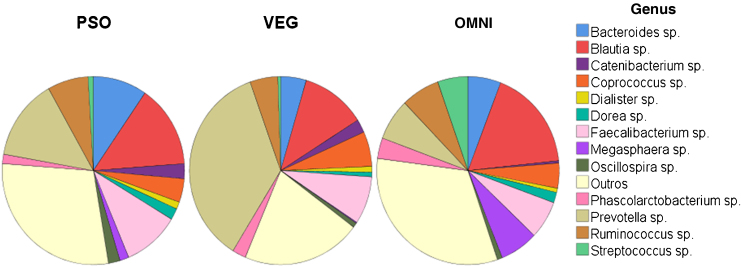
Alpha diversity. Proportion distribution of the 13 most prevalent genera among the groups: PSO (psoriasis), VEG (vegetarian controls), OMNI (omnivore controls)

Regarding alpha-diversity, among the 13 most prevalent genera in the three groups (Fig. 1), a greater proportion of the genus *Prevotella* (36%) was identified in the VEG group and *Blautia* in the PSO (14%) and OMNI (18%) groups.

**Figure 2:**
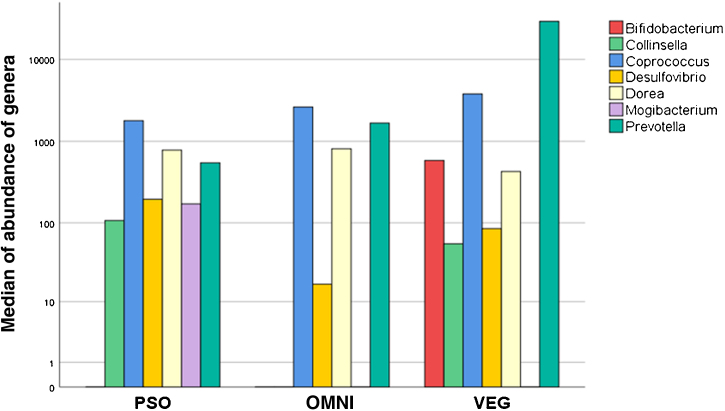
Alpha diversity. Comparison of the median of abundance of genera that showed difference among groups (p < 0.05): PSO (psoriasis), VEG (vegetarian controls), OMNI (omnivore controls)

Regarding the PSO group, there was a difference in the abundance of the genera *Prevotella*, *Mogibacterium*, *Dorea*, *Bifidobacterium* and *Coprococcus*, when compared to the VEG group. The genera *Mogibacterium*, *Collinsella*, *Desulfovibrio*, on the other hand, differed between the PSO and OMNI groups (Fig. 2).

**Figure 3:**
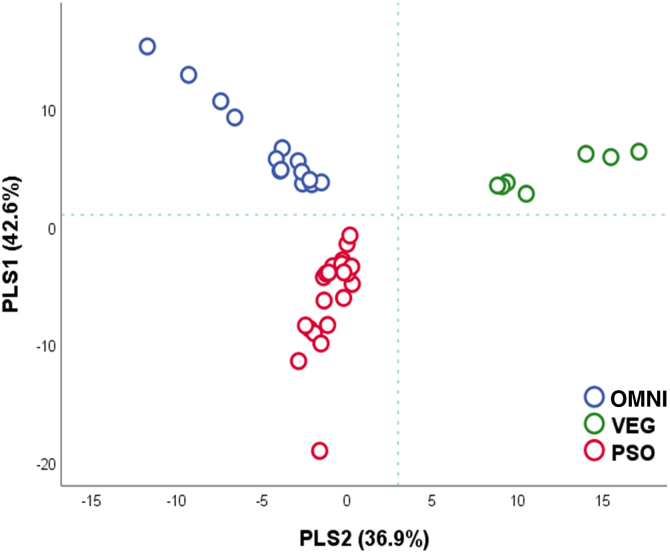
Loading of participants according to PLS components (n = 42). Compositions that discriminate the groups are highlighted. PLS, Partial Least Squares Component; OMNI, Omnivore Controls; VEG, Vegetarian Controls; PSO, Psoriasis

As for beta diversity, two PLS components represented up to 79.5% of the subgroup variance. Fig. 3 depicts the projection of cases according to the component loads, individualizing them in different quadrants. The plsPSO pattern can be understood as the negative loadings of PLS1 and 2 (plsPSO = -PLS1-PS2); conversely, the plsVEG pattern can be represented by the positive loadings of PLS1 and 2 (plsPVEG = PLS1 + PLS2); the plsOMNI pattern can be represented by the positive loading of PS1 and negative loading of PS2 (plsOMNI = PLS1-PLS2).

TU loadings according to PLS1 and 2 are shown in Supplementary Fig. 1. According to the plsPSO components, the bacteria with the highest IM pattern loading in the PSO group were from the genera *Coprococcus*, *Lachnospira*, *Paraprevotella*, and the species *Faecalibacterium prausnitzii* sp., *Prevotella stercorea* sp., *Prevotella copri* sp., and *Ruminococcus callidus* sp. On the other hand, those with the lowest load were from the genera *Lactobacillus*, *Streptococcus*, *Dialister*, *Megasphaera*, Slackia, and the species *Bacteroides fragilis* sp., *Bacteroides uniformis* sp., *Blautia producta* sp., *Butyricicoccus pullicaecorum* sp., and *Parabacteroides distasonis* sp.

**Figure 4:**
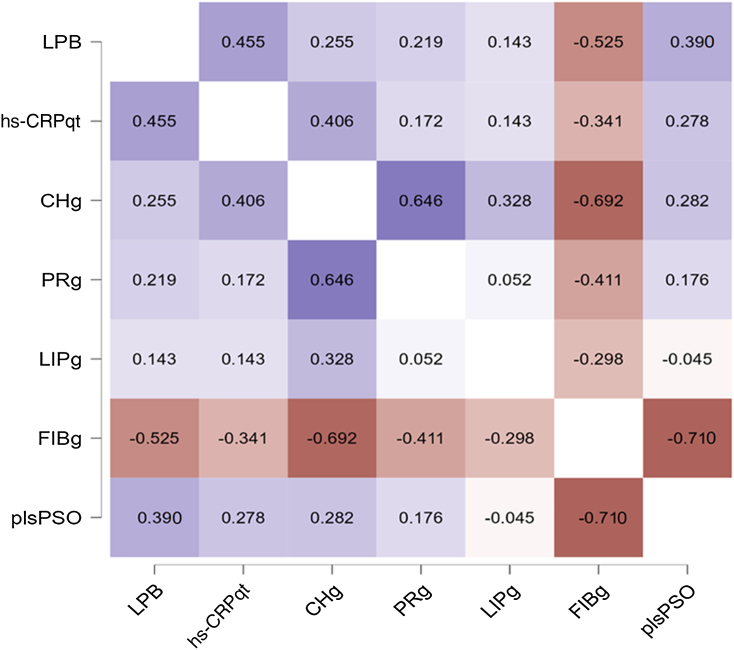
Heat map with bivariate correlations among levels of LPB, CRP, nutritional characteristics of the diet and intestinal microbiome pattern of patients with psoriasis. LPB, Lipopolysaccharide binding protein serum level; hs-CRPqt, highly-sensitive C-reactive protein serum level (quantitative test); CHg, Carbohydrates (in grams); PRg, Proteins (in grams); LIPg, Lipids (in grams); FIBg, Fibers (in grams); plsPSO, PLS component of the intestinal microbiome of participants with psoriasis

Fig. 4 shows the correlations between levels of LPB, hs-CRPqt, nutritional intake and IM patterns: PSO, OMNI, and VEG. LPB levels were inversely correlated with fiber intake (rho = -0.53; p < 0.01), but positively correlated with the IM pattern component of patients with psoriasis (rho = 0.39; p = 0. 02). The behavior of hs-CRPqt was not associated with the plsPSO pattern component (rho = 0.28, p = 0.11), but with carbohydrate intake (rho = 0.41; p = 0.02) and with LPB levels (rho = 0.46; p < 0.01). From the dietary point of view, the IM pattern plsPSO component was strongly and negatively correlated with fiber intake (rho = -0.71; p < 0.01), as well as lower fiber intake was associated with higher levels of LPB and hs-CRPqt.

## Discussion

The present study indicated that IM characteristics of adult men with psoriasis differed from omnivore and vegetarian controls living in the same area of Brazil. Age, dietary, and body composition aspects may also contribute to these differences.^11^

In this sample, the PSO group had a higher intake of carbohydrates; however, with a low fiber intake, showing a higher F/B ratio in IM than the VEG group, which had a higher fiber intake and less abundance of *Firmicutes*. Additionally, the eating habits of patients with psoriasis were unbalanced, as an increase in low-quality fats and high caloric density were also identified, characteristic of the Western diet, which is associated with intestinal dysbiosis and reduced bacterial diversity. These dietary habits may correlate with disease severity and comorbidities, since the consumption of saturated fats, sugars, beef, and alcohol interfere with the systemic inflammatory status, whereas, dietary fiber intake may contribute to the suppression of certain inflammatory pathways and the induction of regulatory T-cells.^12^, ^13^, ^14^

Intestinal dysbiosis also acts on the imbalance of short chain fatty acids (SCFA), of which butyrate and acetate are the most studied ones, produced from fiber fermentation by intestinal bacteria. Acetate is associated with the production of ghrelin, which controls appetite and insulin resistance, while butyrate is a source of energy for intestinal cells, associated with systemic inflammation prevention and reduction and increased bacterial diversity, favoring the proliferation of bacteria of the *Bacteroidetes* phylum. Diets high in fibers, vegetables, and fruits promote an increase in *Bacteroidetes* and, consequently, greater bacterial diversity. On the other hand, diets with an excess of fried foods, sodium, protein, and animal fat may lead to greater systemic pro-inflammatory activation and F/B ratio in IM.^15^ Moreover, SCFA is a direct modulator of IL-17 synthesis by T-lymphocytes.^16^

Intestinal dysbiosis can promote an intraluminal increase in LPS,^17^ and diets with low nutritional quality (e.g., the Western diet), physical and psychological stress, and certain medications (e.g., antimicrobials), are factors that favor increased intestinal permeability. A more permeable intestine favors the translocation of LPS into the bloodstream (metabolic endotoxemia), leading to insulin resistance, adipose tissue inflammation, and tissue infiltration of monocytes/macrophages, which increase the risk of diseases such as hepatic steatosis, obesity, and diabetes. Thus, part of the systemic inflammatory response can be attributed to the composition of IM.^18^, ^19^

In a murine model of imiquimod-induced psoriasis, oral administration of *Staphylococcus aureus* and *Streptococcus danieliae* led to skin inflammation and increased expression of TNF, and IL-17, supporting the hypothesis that intestinal dysbiosis may lead to clinical worsening of the disease.^20^

This study originally investigated associations between plasma levels of LPB, hs-CRPqt, and dietary patterns in omnivores, vegetarians, and patients with psoriasis, demonstrating that LPB levels were inversely correlated with fiber intake. In this sample, participants with psoriasis had higher LPB levels than vegetarians, leading to the hypothesis that IM modulation (e.g., through diet) contributes to reduction in serum LPB levels. Therefore, these findings support that intestinal health can be modulated through food choices. Moreover, hs-CRPqt levels were higher in the PSO group, associated with carbohydrate intake and LPB levels. Although intestinal permeability is not directly assessed, elevated LPB and hs-CRPqt levels may indicate increased intestinal permeability in patients with psoriasis.

Studies with serum levels of LPB in humans are scarce, but there is evidence that LPB is elevated in patients with psoriasis, and correlate with the levels of hs-CRPqt. Thus, LPB may be an indicator of systemic inflammation and progression to metabolic syndrome in patients with psoriasis.^21^ It is noteworthy that LPB has recently been shown to be a biomarker for intestinal permeability in adults, regardless of age, BMI, and sex.^22^

Different findings were obtained in controlled studies that analyzed the IM of patients with psoriasis using different population groups.^23^ In an Israeli study that evaluated beta diversity in patients with psoriasis (n = 24), when compared to controls (n = 46) without psoriasis, matched by age, BMI, and comorbidities, a significant increase was identified in *Ruminococcus gnavus*, *Dorea formicigenerans* and *Collinsella aerofaciens* in the IM of patients with psoriasis, while *Prevotella copri* and *Parabacteroides distasonis* were significantly lower when compared to controls.^24^ This constitutes a somewhat different pattern than the IM composition found in the present sample.

Another study performed in China showed that patients with psoriasis have significantly disordered IM profiles, because when analyzing the IM of 35 patients with psoriasis and 27 controls, a difference was observed regarding the relative abundance of *Firmicutes* and *Bacteroidetes*, with an increase in *Bacteroides*, *Actinobacteria* and a decrease in *Firmicutes*, *Proteobacteria*, and *Bifidobacterium*.^25^ In contrast, another study of patients with psoriasis (n = 19), compared with a healthy control group (n = 20) conducted in Spain, showed lower bacterial diversity and different relative abundance of certain bacterial taxa in patients with psoriasis, with an increase in *Actinobacteria*, *Firmicutes*, and a decrease in *Bacteroidetes*, *Proteobacteria*, *Faecalibacterium*.^26^

In an IM analysis of 52 patients with psoriasis (PASI ≥ 6), compared to a healthy population (n = 300), a higher frequency of the pattern called enterotype 2 was evidenced, with a tendency to show more frequent bacterial translocation and greater inflammatory status (71%) than other enterotypes, with a predominance of *Akkermansia*, *Faecalibacterium*, *Ruminococcus* and a lower proportion of *Bacteroidetes*.^27^ The present sample also indicated higher proportions of *Faecalibacterium* and *Ruminococcus*, in addition to lower levels of *Bacteroidetes* in the profile (PLS) of patients with psoriasis.

Recently, a study conducted in Argentina demonstrated changes in IM composition depending on psoriasis status, with increased *Firmicutes* and depletion of *Bacteroidetes* in 55 patients with psoriasis when compared to 27 controls. Additionally, the *Faecalibacterium* and *Blautia* genera were higher in patients with psoriasis, while *Bacteroides* and *Paraprevotella* were higher in controls without psoriasis.^28^ Since the patients in the present study were undergoing treatment, and most of them had PASI < 4, it was not possible to stratify the IM according to disease severity.

Another case-control study in Central Asia, with patients aged between 30 and 45 years, with psoriasis (n = 20) and controls (n = 20), matched for age and sex, found an association between psoriasis and elevated levels of *Firmicutes*, *Faecalibacterium* and reduced abundance of *Oscillibacter* and *Roseburia*, but there was no difference regarding diversity and the F/B ratio, very much in line with the results of the present study.^29^

A Brazilian study showed less diversity in the IM of women with psoriasis (n = 21) when compared to controls (n = 24), identifying an increase in the *Dialister* genus and the *Prevotella copri* sp., with a reduction in *Ruminococcus*, *Lachnospira*, and *Blautia* genera, and *Akkermansia muciniphila* sp.^30^

In the present study, the IM profile found in adult men with psoriasis was associated with those described in other comorbidities, such as metabolic syndrome, obesity, and affective diseases, which may be due to the Western lifestyle. Also according to the literature, *Coprococcus* was associated with affective diseases such as depression, obesity, and poor sleep quality^31^; *Lachnospira* was associated with the development of diabetes mellitus and *Paraprevotella* with higher fat intake.^33^ Although there is an association with vegetarian diets, *Prevotella* is also associated with high carbohydrate intake, and as it has high genetic diversity within and between species, this may explain its description in healthy individuals IM and in those with dysbiosis; and a high load of *Prevotella* in IM has been associated with obesity, insulin resistance, hypertension, and non-alcoholic fatty liver disease.^34^

The composition of IM in patients with psoriasis found in the present sample was opposed to that found in vegetarians, rich in *Bacteroides* and *Lactobacillus*, associated with lower intestinal permeability. In fact, the prevalence of psoriasis in vegetarians is rare, and the vegetarian diet leads to improvement in psoriatic skin lesions, suggesting that IM modulation may support the development of inflammatory diseases such as psoriasis.^35^, ^36^

Metagenomic studies of IM show high variability of results. Ethnic, cultural, and dietary differences between populations may explain the controversial results of studies that characterized IM in patients with psoriasis.

Thus, what constitutes a healthy IM has yet to be defined, after excluding known pathogenic bacteria. Therefore, it is essential to emphasize that commensal species should not be considered absolutely “good” or “bad” since they all play a role in the intestinal ecosystem. The problem emerges from the imbalance, that is, the predominance of groups of commensal species that promote changes in intestinal permeability. Moreover, when characterizing the IM of a group, the aim is to understand the imbalance pattern, although the IM composition is unique in each individual, is acquired in the first years of life and shaped by environmental characteristics, such as eating habits, throughout life.^37^, ^38^, ^39^

The present study has limitations related to the modest, non-randomized sample, which did not prevent the identification of IM patterns among the groups studied. Moreover, the common origin of the population group maximizes sample homogeneity. The effect of psoriasis treatments on IM is a topic to be investigated and the participants used several different drugs; however, the use of adalimumab was not shown to modify the IM pattern in ten patients.^40^ The participants of the present study also had low PASI scores, due to disease control with the established therapy. In fact, IM studies with patients with psoriasis without systemic treatment are needed. Another aspect refers to the sampling of the fecal microbiome, which consists of the transient flora of the colon, in contrast to the permanent flora that is related to the mucosa, less subject to variation. Finally, LPB is used as a proxy for LPS measurement, due to its lability. Dietary variations can make comparisons among groups difficult since even among the omnivore, vegetarian, and psoriasis groups, there are foods that can act differently on the IM.

Controlled clinical studies in psoriasis aimed at manipulating the IM, whether with stool transplantation, dietary interventions (e.g., fiber intake adequacy), pro/prebiotics, or bile salts, must be carried out to better understand the role of the IM in the clinical activity of psoriasis.

## Conclusion

A difference was identified in the IM of Brazilian adult men with psoriasis, in comparison to healthy omnivores and vegetarian controls. The identified IM pattern correlated with dietary fiber intake and LPB serum levels. Changes in eating habits can remodel IM, and consequently help in the clinical control of psoriasis. The present study emphasizes the need to raise awareness of the nutritional importance in the treatment of the disease and the management of comorbidities.

## Financial support

FUNADERM - Fundo de Apoio à Dermatologia, da Sociedade Brasileira de Dermatologia.

## Authors' contributions

Hélio Amante Miot: Design of the study; analysis of results; drafting of the manuscript; review and approval of the final version of the manuscript.

Tatiana Cristina Figueira Polo: Design of the study; collection of data; analysis of results; drafting of the manuscript; review and approval of the final version of the manuscript.

Mariana Righetto de Ré Lai: Design of the study; data collection; analysis of results; drafting of the manuscript; review and approval of the final version of the manuscript.

Luciane Donida Bartoli Miot: Data collection; review and approval of the final version of the manuscript.

Giovana Fernanda Cosi Bento: Analysis of results; review and approval of the final version of the manuscript.

Márcia Guimarães da Silva: Review and approval of the final version of the manuscript.

Silvio Alencar Marques: Review and approval of the final version of the manuscript.

## Conflicts of interest

None declared.

## Figures and Tables

**Figure:**