# Supplementary material for: Intestinal microbiome characterization of adult Brazilian men with psoriasis compared to omnivore and vegetarian controls
Source: An Bras Dermatol. 2023 May 6;98(5):635–43. doi: 10.1016/j.abd.2022.08.008 (PMC10404490; doi:10.1016/j.abd.2022.08.008)
Supplement: Supplementary file 1 [file mmc2.docx]

**ABD-D-22-00225_Supplementary Material**

**Supplementary Table 1** Taxonomic units (TU = 1,496), distributed in phyla, classes, orders, families, genera and species.

| **Phylum (12)** | **Class (22)** | **Order (31)** | **Family (52)** | **Genus (94)** | **Species (64)** |
| --- | --- | --- | --- | --- | --- |
| Firmicutes | Clostridia | Clostridiales | Ruminococcaceae | Prevotella | *Prevotella copri* |
| Bacteroidetes | Bacteroidia | Bacteroidales | Lachnospiraceae | Bacteroides | *Faecalibacterium prausnitzii* |
| Actinobacteria | Coriobacteriia | Coriobacteriales | Prevotellaceae | Ruminococcus | *Prevotella stercorea* |
| Proteobacteria | Erysipelotrichi | Erysipelotrichales | Coriobacteriaceae | Oscillospira | *[Ruminococcus] gnavus* |
| Tenericutes | Bacilli | RF39 | Bacteroidaceae | Blautia | *Parabacteroides distasonis* |
| Cyanobacteria | Mollicutes | Lactobacillales | Erysipelotrichaceae | Coprococcus | *Blautia producta* |
| Lentisphaerae | Betaproteobacteria | Burkholderiales | Veillonellaceae | Faecalibacterium | *Bacteroides uniformis* |
| Synergistetes | Gammaproteobacteria | Desulfovibrionales | [Paraprevotellaceae] | [Ruminococcus] | *[Eubacterium] biforme* |
| Euryarchaeota | Deltaproteobacteria | Enterobacteriales | [Odoribacteraceae] | Butyricimonas | [Eubacterium] dolichum |
| Fusobacteria | Actinobacteria | Actinomycetales | Christensenellaceae | Parabacteroides | Bacteroides plebeius |
| Verrucomicrobia | Alphaproteobacteria | YS2 | [Mogibacteriaceae] | Sutterella | Bacteroides ovatus |
| Elusimicrobia | 4C0d-2 | RF32 | S24-7 | Catenibacterium | Blautia obeum |
|  | [Lentisphaeria] | Victivallales | Porphyromonadaceae | Dorea | Coprococcus eutactus |
|  | Synergistia | Synergistales | Desulfovibrionaceae | [Prevotella] | Butyricicoccus pullicaecorum |
|  | Fusobacteriia | Fusobacteriales | Rikenellaceae | Paraprevotella | *Alistipes indistinctus* |
|  | Methanobacteria | Aeromonadales | Alcaligenaceae | [Eubacterium] | *Ruminococcus callidus* |
|  | Opitutae | Methanobacteriales | Streptococcaceae | Megasphaera | *Lactobacillus ruminis* |
|  | Verrucomicrobiae | Bifidobacteriales | [Barnesiellaceae] | Streptococcus | *Bacteroides eggerthii* |
|  | RF3 | [Cerasicoccales] | Lactobacillaceae | Lactobacillus | *Collinsella stercoris* |
|  | Epsilonproteobacteria | Bacillales | Enterobacteriaceae | Roseburia | *Ruminococcus bromii* |
|  | Thermoplasmata | Turicibacterales | Clostridiaceae | Collinsella | *Clostridium spiroforme* |
|  | Elusimicrobia | Verrucomicrobiales | Actinomycetaceae | Dialister | *Ruminococcus flavefaciens* |
|  |  | ML615J-28 | Peptococcaceae | Lachnospira | *Bacteroides coprophilus* |
|  |  | Campylobacterales | Dehalobacteriaceae | Slackia | *Akkermansia muciniphila* |
|  |  | Anaeroplasmatales | Victivallaceae | Clostridium | *Bacteroides fragilis* |
|  |  | Elusimicrobiales | Fusobacteriaceae | Odoribacter | *Bacteroides caccae* |
|  |  | E2 | Succinivibrionaceae | Desulfovibrio | *Streptococcus anginosus* |
|  |  | SHA-98 | Synergistaceae | Phascolarctobacterium | *Coprococcus catus* |
|  |  | Neisseriales | Methanobacteriaceae | Dehalobacterium | *Eggerthella lenta* |
|  |  | Pasteurellales | [Tissierellaceae] | Acidaminococcus | *Bulleidia p-1630-c5* |
|  |  | Gemellales | Bifidobacteriaceae | Actinomyces | *Desulfovibrio D168* |
|  |  |  | [Cerasicoccaceae] | Bulleidia | *Dorea formicigenerans* |
|  |  |  | Leuconostocaceae | Christensenella | *Veillonella dispar* |
|  |  |  | Helicobacteraceae | Alistipes | *Clostridium hathewayi* |
|  |  |  | Eubacteriaceae | Adlercreutzia | *Collinsella aerofaciens* |
|  |  |  | Oxalobacteraceae | Bilophila | *Pyramidobacter piscolens* |
|  |  |  | Turicibacteraceae | Butyricicoccus | *Streptococcus agalactiae* |
|  |  |  | Micrococcaceae | Succinivibrio | *Clostridium symbiosum* |
|  |  |  | Verrucomicrobiaceae | Anaerostipes | *Butyrivibrio crossotus* |
|  |  |  | Pasteurellaceae | Methanobrevibacter | *Bacillus halodurans* |
|  |  |  | Staphylococcaceae | Mitsuokella | *Clostridium butyricum* |
|  |  |  | Aerococcaceae | Megamonas | *Rothia mucilaginosa* |
|  |  |  | Bacillaceae | Holdemania | *Clostridium colicanis* |
|  |  |  | Gemellaceae | Lactococcus | *Bulleidia moorei* |
|  |  |  | Dethiosulfovibrionaceae | AF12 | *[Ruminococcus] torques* |
|  |  |  | EtOH8 | Anaerofilum | *Streptococcus sobrinus* |
|  |  |  | Elusimicrobiaceae | Oribacterium | *Clostridium hiranonis* |
|  |  |  | [Methanomassiliicoccaceae] | Coprobacillus | *[Eubacterium] cylindroides* |
|  |  |  | Neisseriaceae | Synergistes | *Lactobacillus delbrueckii* |
|  |  |  | Corynebacteriaceae | Mogibacterium | *Roseburia inulinivorans* |
|  |  |  | Peptostreptococcaceae | Fusobacterium | *Lactobacillus helveticus* |
|  |  |  | Anaeroplasmataceae | Leuconostoc | *Ruminococcus albus* |
|  |  |  |  | Lachnobacterium | *Lactobacillus mucosae* |
|  |  |  |  | Veillonella | *Alistipes putredinis* |
|  |  |  |  | Bifidobacterium | *Clostridium lavalense* |
|  |  |  |  | Atopobium | *Ruminococcus gauvreauii* |
|  |  |  |  | Anaerotruncus | *Lactobacillus zeae* |
|  |  |  |  | Butyrivibrio | *Coprobacillus cateniformis* |
|  |  |  |  | Rc4-4 | *Lactococcus garvieae* |
|  |  |  |  | Eggerthella | *Succinatimonas hippei* |
|  |  |  |  | Cloacibacillus | *Clostridium perfringens* |
|  |  |  |  | Parvimonas | *Victivallis vadensis* |
|  |  |  |  | Akkermansia | *Clostridium ramosum* |
|  |  |  |  | Peptococcus | *Alistipes massiliensis* |
|  |  |  |  | Turicibacter |  |
|  |  |  |  | Helicobacter |  |
|  |  |  |  | Porphyromonas |  |
|  |  |  |  | Pseudoramibacter Eubacterium |  |
|  |  |  |  | SMB53 |  |
|  |  |  |  | VadinCA11 |  |
|  |  |  |  | Alloscardovia |  |
|  |  |  |  | Neisseria |  |
|  |  |  |  | Peptoniphilus |  |
|  |  |  |  | Nesterenkonia |  |
|  |  |  |  | Finegoldia |  |
|  |  |  |  | Anaerococcus |  |
|  |  |  |  | Corynebacterium |  |
|  |  |  |  | Proteus |  |
|  |  |  |  | Victivallis |  |
|  |  |  |  | Anaerovibrio |  |
|  |  |  |  | Oxalobacter |  |
|  |  |  |  | Pyramidobacter |  |
|  |  |  |  | Staphylococcus |  |
|  |  |  |  | Methanosphaera |  |
|  |  |  |  | Succinatimonas |  |
|  |  |  |  | RFN20 |  |
|  |  |  |  | CF231 |  |
|  |  |  |  | Epulopiscium |  |
|  |  |  |  | Bacillus |  |
|  |  |  |  | Rothia |  |
|  |  |  |  | Moryella |  |
|  |  |  |  | Cc_115 |  |
|  |  |  |  | Sarcina |  |
|  |  |  |  | Scardovia |  |

**Supplementary Figure 1** TU dispersion according to PLS1 and PLS2 loads.
